# Telomerase deficiency impairs glucose metabolism and insulin secretion

**DOI:** 10.18632/aging.100200

**Published:** 2010-09-14

**Authors:** Doreen Kuhlow, Simone Florian, Guido von Figura, Sandra Weimer, Nadja Schulz, Klaus J. Petzke, Kim Zarse, Andreas F.H Pfeiffer, K. Lenhard Rudolph, Michael Ristow

**Affiliations:** ^1^ Department of Human Nutrition, Inst. of Nutrition, University of Jena, Jena, D-07743, Germany; ^2^ Department. of Clinical Nutrition, German Institute of Human Nutrition Potsdam-Rehbrücke, Nuthetal, D-14558, Germany; ^3^ Department of Nutritional Toxicology, German Institute of Human Nutrition Potsdam-Rehbrücke, Nuthetal, D-14558, Germany; ^4^ Department of Molecular Medicine, University of Ulm, Ulm, D-89081, Germany; ^5^ Department of Internal Medicine I, University of Ulm, Ulm, D-89081, Germany; ^6^ Department of Experimental Diabetology, German Institute of Human Nutrition Potsdam-Rehbrücke, Nuthetal, D-14558, Germany; ^7^ Stable Isotope Group, German Institute of Human Nutrition Potsdam-Rehbrücke, Nuthetal, D-14558, Germany; ^8^ Department of Endocrinology, Diabetes and Nutrition, Charité University Medicine, CBF, Berlin, D-12203, Germany; ^9^ Max-Planck-Research Group on Stem Cell Aging, University of Ulm, Ulm, D-89081, Germany

**Keywords:** Telomere, telomerase, senescence, diabetes mellitus, glucose intolerance, Insulin secretion, beta-cell

## Abstract

Reduced telomere length and impaired telomerase activity have been linked to several diseases associated with senescence and aging. However, a causal link to metabolic disorders and in particular diabetes mellitus is pending. We here show that young adult mice which are deficient for the *Terc* subunit of telomerase exhibit impaired glucose tolerance. This is caused by impaired glucose-stimulated insulin secretion (GSIS) from pancreatic islets, while body fat content, energy expenditure and insulin sensitivity were found to be unaltered. The impaired secretion capacity for insulin is due to reduced islet size which is linked to an impaired replication capacity of insulin-producing beta-cells in *Terc*-deficient mice. Taken together, telomerase deficiency and hence short telomeres impair replicative capacity of pancreatic beta-cells to cause impaired insulin secretion and glucose intolerance, mechanistically defining diabetes mellitus as an aging-associated disorder.

## INTRODUCTION

Diabetes mellitus is the most prevalent metabolic disorder, and its metabolic hallmark, glucose intolerance, affects approximately 344 million people worldwide [1]. There are two prominent subtypes of diabetes mellitus, named type 1 and type 2, respectively. Both sub-types affect the production and secretion of insulin from pancreatic beta-cells. While type 1 diabetes is considered an autoimmune disease leading to slowly progressive and ultimately complete loss of pancreatic beta-cells, type 2 diabetes is caused by a combination of peripheral insulin resistance and beta-cell dysfunction. Recent evidence however suggests that type 2 diabetes is additionally characterized by impaired beta-cell regeneration and reduced beta-cell mass [2-5].

Eukaryotic chromosomes carry tandemly repeated terminal sequences, so-called telomeres, which are essential for chromosome stability. Telomeric DNA is synthesized, elongated and hence maintained by copying an RNA template sequence within the RNA moiety of an enzyme called telomerase. The respective subunit of telomerase is encoded by the *telomerase RNA component* (*Terc*) gene. Significant evidence suggests that telomeres and telomerase activity have a crucial role in regulating cell survival and regeneration [6-8].

Given the role of telomerase in maintenance and replication of differentiated cells on the one hand, and the importance of beta-cell loss for the pathogenesis of type 1 as well as type 2 diabetes on the other hand, it appears tempting to hypothesize that telomerase could act as key regulator of beta-cell viability and regeneration. We therefore here have analyzed previously generated mice [9,10] that are deficient for the *telomerase RNA component* (*Terc*) gene in this regard. We find that these animals develop glucose intolerance and impaired insulin secretion due to impaired beta-cell regeneration.

## RESULTS

### Body composition and energy expenditure in *Terc-/- G4* mice

**Figure 1.**
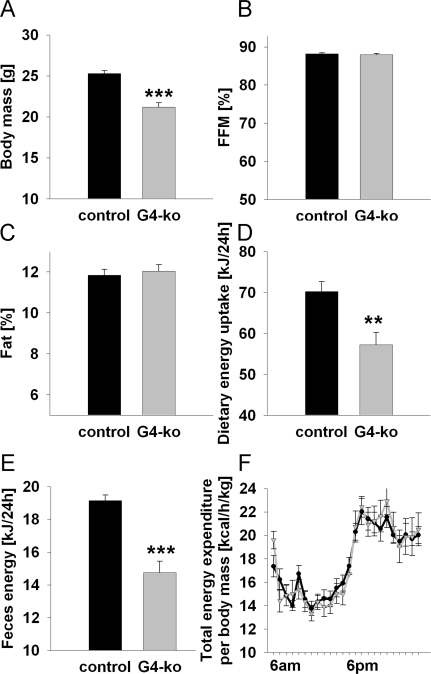
Impaired telomerase activity - Effects on body mass, body composition and energy expenditure. (**A**) Depicts body mass of 19-24 week old *Terc-/-* G4 animals (grey) and corresponding controls (black) (n=60 controls, n=61 *Terc-/-* G4). (**B**) Relative fat-free mass and (**C**) relative body fat content in mice as in Panel A (n=60 controls, n=61 *Terc-/-* G4). (**D**) Food uptake per individual mouse, depicted for mice as in Panel A (n=30 controls, n=10 *Terc-/-* G4). (**E**) Energy excreted via faeces as quantified by direct bomb calorimetry (n=30 controls, n=10 *Terc-/-* G4). (**F**) Total energy expenditure normalized to body mass of mice as in Panel A (n=10 controls, n=10 *Terc-/-* G4).

As previously reported [9,10], *Terc -/-* G4 mice are smaller and have reduced body mass (Figure 1A). However, we here show that this reduction affects both lean mass (Figure 1B) and body fat (Figure 1C) to the same extent. We moreover demonstrate that both dietary energy uptake (Figure 1D) and energy excretion via *faeces* (Figure 1E) is reduced. However when these latter findings are normalized to body mass, no significant differences can be found for neither energy uptake nor energy excretion (data not shown). Furthermore, no glucosuria was detected in none of the genotypes (data not shown), precluding differential loss of energy via urine. Accordingly determination of energy expenditure as quantified by indirect calorimetry did not show any differences regarding energy turnover at neither day- nor night-time (Figure 1F).

Taken together these findings indicate that *Terc -/-* G4 mice are smaller but have an unaltered relative body composition and energy metabolism.

### Metabolic plasma markers in *Terc -/-* G4 mice

We quantified concentrations of glucose (fasted and fed states), insulin (fasted and fed states), triglycerides, non-esterified fatty acids, total cholesterol and alanine aminotransferase (all fasted) in plasma samples of the animals, and found no differences between *Terc -/-* G4 and control genotypes (Suppl. Figure 1).

### Glucose metabolism in *Terc -/-* G4 mice

**Figure 2.**
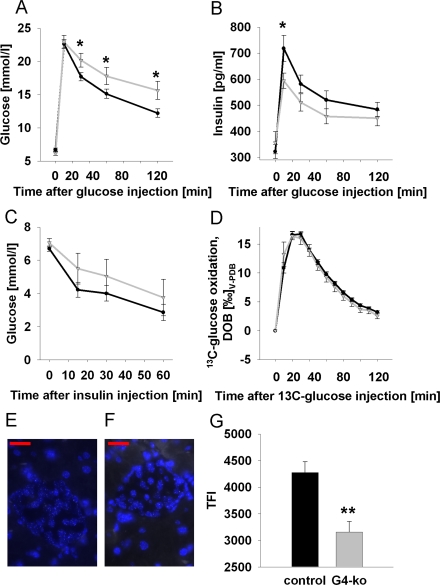
Impaired telomerase activity impairs glucose tolerance and glucose-stimulated insulin secretion. (**A**) Serum glucose excursions following intraperitoneal injection of D-glucose; black line corresponds to control animals while grey line reflects *Terc-/-* G4 animals (also applies to panels B-D) (n=47 controls, n=25 *Terc-/-* G4). (**B**) Plasma insulin excursions following intraperitoneal injection of D-glucose (n=47 controls, n=25 *Terc-/-* G4). (**C**) Blood glucose excursion following intraperitoneal injection of insulin (n=24 controls, n=8 *Terc-/-* G4). (**D**) Exhalation of ^13^Carbon dioxide per constant time interval following intraperitoneal injection of ^13^C-labeled glucose (n=23 controls, n=8 *Terc-/-* G4). Typical quantitative fluorescence in situ hybridization (qFISH) appearances of a control (**E**) and Terc -/- G4 **(F)** islet, respectively. (**G**) depicts results of telomere length quantification by qFISH (n=23 control and n=10 *Terc-/-* G4 animals, 25 *nuclei* per mouse were evaluated; red bars are 20 μm of length).

We next performed intra-peritoneal glucose tolerance tests (ipGTT) on *Terc -/-* G4 and on the control genotypes, and observed impaired glucose clearing in *Terc -/-* G4 mice (Figure 2A). We then aimed to elucidate whether impaired insulin secretion or rather insulin resistance may be responsible for this phenotype. We therefore first quantified plasma insulin levels during conditions of ipGTT, and observed impaired insulin secretion in response to injected glucose in *Terc -/-* G4 mice (Figure 2B). By contrast, performing intra-peritoneal insulin tolerance tests (ipITT) by injecting insulin into mice did not reveal significant differences in glucose disposal in response to insulin (Figure 2C). Since telomerase has been reported to affect mitochondrial function [11,12], we lastly quantified glucose oxidation rates by injecting uniformly ^13^C-labeled glucose to quantify the exhalation of ^13^CO_2_, which was found to be identical in all genotypes (Figure 2D), precluding systemic mitochondrial dysfunction in *Terc -/-* G4 mice, at least in regards to glucose-derived pyruvate oxidation capacity.

Since the phenotype appeared to be restricted to impaired insulin secretion, we next quantified telomere length specifically in pancreatic islets. Using quantitative fluorescence in situ hybridization (qFISH) (Figure 2 E, F) we observe a pronounced reduction of average telomere length in islets of *Terc -/-* G4 animals (Figure 2G).

Taken together, telomerase deficiency causes shortened telomere length in pancreatic islets, and impaired glucose tolerance as well as impaired insulin secretion in mice.

### Reduced beta-cell mass in *Terc -/-* G4 mice

**Figure 3.**
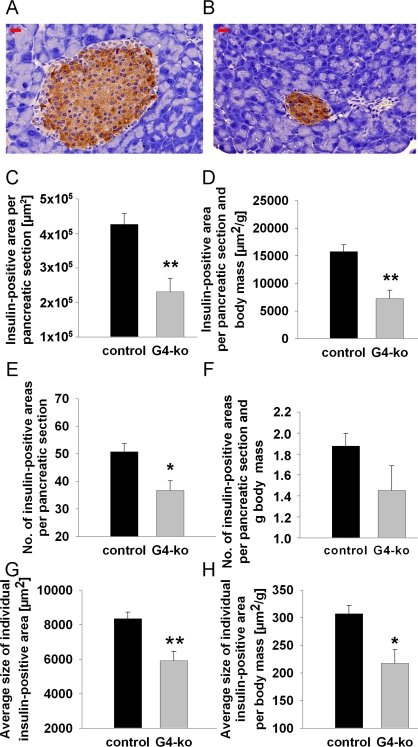
Impaired telomerase activity reduces number and size of insulin-containing pancreatic islets. (**A**) Depicts a typical control islet, while (**B**) reflects a typical islet from an *Terc-/-* G4 animal; red bars are 20 μm of length. (**C, D**) depict insulin-positive area size normalized to whole pancreatic section area only (**C**) and additionally normalized to body mass (**D**). (**E, F**) shows the number of islets per pancreatic section (**E**) and after additional normalization to body mass (**F**). (**G, H**) depict the sizes of individual insulin-positive areas *per se* (**G**) and after normalization to body mass **(H)**. Black bars reflect control genotypes (n=25) and grey bars indicate *Terc-/-* G4 animals (n=10).

Reduced insulin secretion is typically caused by reduced mass of the islets of Langerhans, which mainly contain beta cells. To decide whether this possibility may apply in regards to the phenotype observed (Figure 2 A, B), we first determined beta-cell mass in repeated sections from *pancreata* of all four genotypes by quantitative microscopy. We firstly observed smaller islets in mice deficient for Terc (Figure 3B) as compared to control animals (Figure 3A). By morphometrically quantifying insulin-positive areas within pancreatic sections of all genotypes, we consistently found a reduction of insulin-positive areas per total pancreatic section (Figure 3C). Since *Terc -/-* G4 are smaller than control genotypes (Figure 1A), we normalized pancreatic and insulin-positive areas to body mass to exclude the possibility that reduced insulin-positive area size simply was an indicator of reduced body mass. After normalizing the areas for body mass, we still observe a significant reduction of insulin-positive areas (Figure 3D).

We moreover find the number of islets to be reduced in *Terc -/-* G4 animals (Figure 3E) which however did not apply after normalization for body mass (Figure 3F, P=n.s.). Most importantly, we find the insulin-positive area per individual islet of Langerhans to be reduced in absolute terms (Figure 3G) as well as after normalization for body mass (Figure 3H), altogether suggesting that telomerase deficiency causes a reduction of beta cell mass by reducing the size of individual islets whereas islet number is not affected.

### Impaired beta-cell regeneration in *Terc -/-* G4 mice

**Figure 4.**
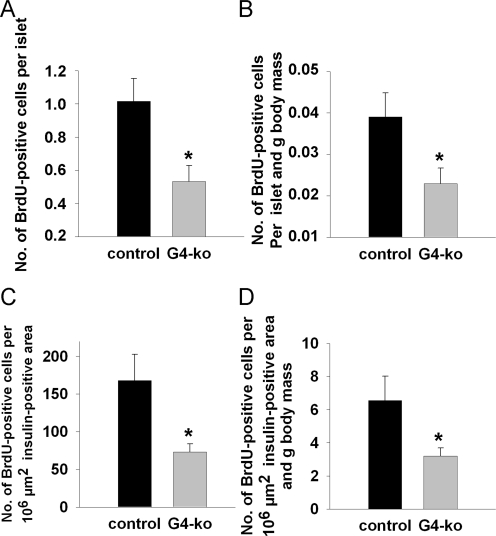
Impaired telomerase activity reduces islet number and size by impairing the replicative potential of pancreatic beta-cells. (**A, B**) shows the number of BrdU-positive cells per insulin-positive area **(A)** and after additional normalization to body mass (**B**). (**C, D**) depict the number of BrdU-positive cells per standardized individual insulin-positive area (**C**) and after additional normalization to body mass (**D**). Black bars reflect control genotypes (n=9) and grey bars indicate *Terc-/-* G4 animals (n=10).

We next injected bromo-desoxyuridine (BrdU) into mice prior to sacrifice which enables us to determine the number of DNA-replicating, *i.e.* dividing beta cells, after counterstaining with an anti-BrdU antibody. We found a reduction of BrdU-positive beta-cells per islet (Figure 4A) as well as after normalization for body mass (Figure 4B). Lastly, the number of BrdU-positive beta-cells per standardized insulin-positive area was also found to be reduced (Figure 4C) as well as after normalization for body mass (Figure 4D).

Taken together, these findings indicate that telomerase deficiency as found in *Terc -/-* G4 animals causes reduced beta-cell mass due to impaired regenerative capacity of pancreatic islets of Langerhans, culminating in impaired glucose tolerance due to reduced insulin secretion *in vivo*.

## DISCUSSION

We here describe a direct causal link between impaired telomerase activity and impaired insulin secretion as well as glucose intolerance in *Terc-/-* G4 mice. These findings are supported by the fact that shortened telomeres, as found in states of reduced telomerase activity, are associated with type 2 [13-20] as well as possibly type 1 diabetes mellitus [21,22] in humans. As stated in the introductory section, both disorders exhibit patterns of impaired insulin secretion.

According to our findings, glucose intolerance in *Terc-/-* G4 animals is caused by reduced insulin secretion only, whereas insulin sensitivity was found to be unaffected. This latter observation is in conflict with previous reports that shortened telomeres in humans are associated with insulin resistance [14,23-25], a state in which insulin sensitivity is reduced. However and since insulin secretion and insulin sensitivity appear to influence each other primary disturbances in insulin secretion may secondarily affect insulin resistance.

Progressive beta-cell failure and senescence are hallmarks of type 2 diabetes [2-5]. More specifically, telomere shortening has been shown to determine the risk of beta-cell growth arrest and cellular senescence in adult human islet cells [26]. The *Tert*gene is moderately expressed in islets from different mouse models, including C57BL/6 (the strain used in the present study), and expression levels interestingly appear to decrease with age [27]. Moreover, significant telomerase activity can be detected in islet extracts from C57BL/6 mice [27]. Interestingly, telomerase appears to counteract the senescence-promoting effects of hyperglycemia, at least *in vitro* [28]. Moreover, several of the known downstream targets of telomerase have been identified to affect islet cell growth and regeneration [29,30], whereas a link to telomerase deficiency had not been established. Further studies will have to elucidate whether our current findings and these putative downstream effectors converge in deterioration of islet mass and hence development of diabetes mellitus.

Taken together, our findings indicate that reduced telomerase activity may be considered a cause of impaired glucose tolerance and hence diabetes mellitus. In full accordance with the role of this protein in multiple other cell types, telomerase is required to maintain the replicative potential of pancreatic beta-cells. Consistently, lack of telomerase activity causes beta-cell loss and subsequent deterioration of glucose metabolism, reflecting the increasing incidence of diabetes mellitus with increasing age.

## MATERIALS AND METHODS

### Generation and maintenance of mice

Four genotypes of the previously described *Terc -/-* animals were studied: While *Terc -/-* G4 mice have been intercrossed for three generations with Terc-deficient littermates, *Terc-/-* G1 have parents that were both heterozygous for the *Terc* disruption. The latter served as controls, in addition to wild-type and heterozygous *Terc +/-* animals, since significant telomere shortening is absent in such *Terc -/-* G1 animals [9,10]. Tail-biopsy genomic PCR for detection of the *Terc-/-* allele was performed as previously described [10]. Animals were housed in air-conditioned rooms (temperature, 20 ± 2 °C; relative moisture, 50 - 60 %) under a 12-hour-light/dark schedule (lights on at 06:00 hrs) and had free access to standard chow (*Ssniff R/M-H*, Ssniff Spezialdiäten GmbH, Soest, Germany) and water. Mice were studied at an age of 19 - 24 weeks, and were kept in accordance with the National Institutes of Health guidelines for the care and use of laboratory animals, and all experiments were approved the corresponding institutional review boards.

### Body composition analysis

Body mass and body composition (including fat content) were measured by use of quantitative nuclear magnetic resonance technique (Echo MRI-100 Body Composition Analyzer, Echo Medical Systems, Houston, TX) as previously described [31].

### Food consumption quantification

Food intake was recorded weekly and *faeces* were collected 3 times per week. After freeze-drying energy content of diet and *faeces* samples was determined by bomb calorimetry (IKA C5003, IKA Werke GmbH, Staufen, Germany) as previously described [31].

### Respiratory quotient and energy expenditure

Total energy expenditure and respiratory quotient were determined by indirect calorimetry of mice housed individually in metabolic cages, receiving food and water *ad libitum* as described using a TSE LabMaster Calorimetry Module (TSE, Bad Homburg, Germany) [31].

### Insulin and glucose tolerance tests

Glucose tolerance tests were performed as described [31] by intraperitoneal (ip) glucose injection of 1 g per kg body mass of D-(+)-glucose (Merck, Darmstadt, Germany) after mice were fasted 16 hrs overnight. Insulin tolerance tests were performed by intraperitoneal (ip) injection of 1 U insulin per kg body mass as described [32].

### Plasma and serum analyses

All blood samples were obtained, prepared, stored and measured exactly as previously described [31].

### ^13^CO_2_ breath tests for glucose oxidation experiments

^13^CO_2_ breath tests were applied as non-invasive methods to study the oxidation of injected or ingested ^13^C-labeled glucose exactly as previously described [31].

### Quantification of pancreas and islet sizes

All procedures, including immuno-histochemistry for insulin, BrdU-injection and detection were performed as described [32] while digitizing and quantification for beta-cell-mass was performed with a MiraxMidi slide scanner and the AutMess program of AxioVision 4.6 software (both from Carl Zeiss MicroImaging, Jena, Germany).

### Telomere length

in pancreatic islets was determined on sections as above using quantitative fluorescence in situ hybridization (qFISH) according to previously reported methods [33]. The relative telomere length was determined by the fluorescence intensity using the TFL software package [34].

### Data analysis

Data are expressed as mean ± SEM. Number of animals analyzed is given in the respective figure legends. Equal distributions were tested by Kolmogorov-Smirnov test before applying T-tests. Unpaired Student's T-tests were used to compare *Terc-/-* G4, *Terc -/-* G1, *Terc +/-* and wild-type animals by employing SPSS 15.0 (SPSS, Chicago, IL, USA).

## SUPPLEMENTAL MATERIAL

Effects of impaired telomerase activity on metabolic serum markers. (**A, B**) Fasting plasma glucose (**A**) and insulin (**B**) levels of mice that were food deprived for 16 hrs (**C, D**) Postprandial plasma glucose (**C**) and insulin (**D**) levels of mice that had ad libitum access to diet over night. (**E** to **H**) Fasting serum triglyceride (**E**), non-esterified fatty acid (**F**), total cholesterol (**G**) and alanine aminotransferase (ALAT) (**H**) levels. Black bars reflect control genotypes (n=42) and grey bars indicate Terc-/- G4 animals (n=19).
