# Supplementary figures and images for: Telomerase deficiency impairs glucose metabolism and insulin secretion
Source: Aging (Albany NY). 2010 Sep 14;2(10):650–8. doi: 10.18632/aging.100200 (PMC2993795; doi:10.18632/aging.100200)

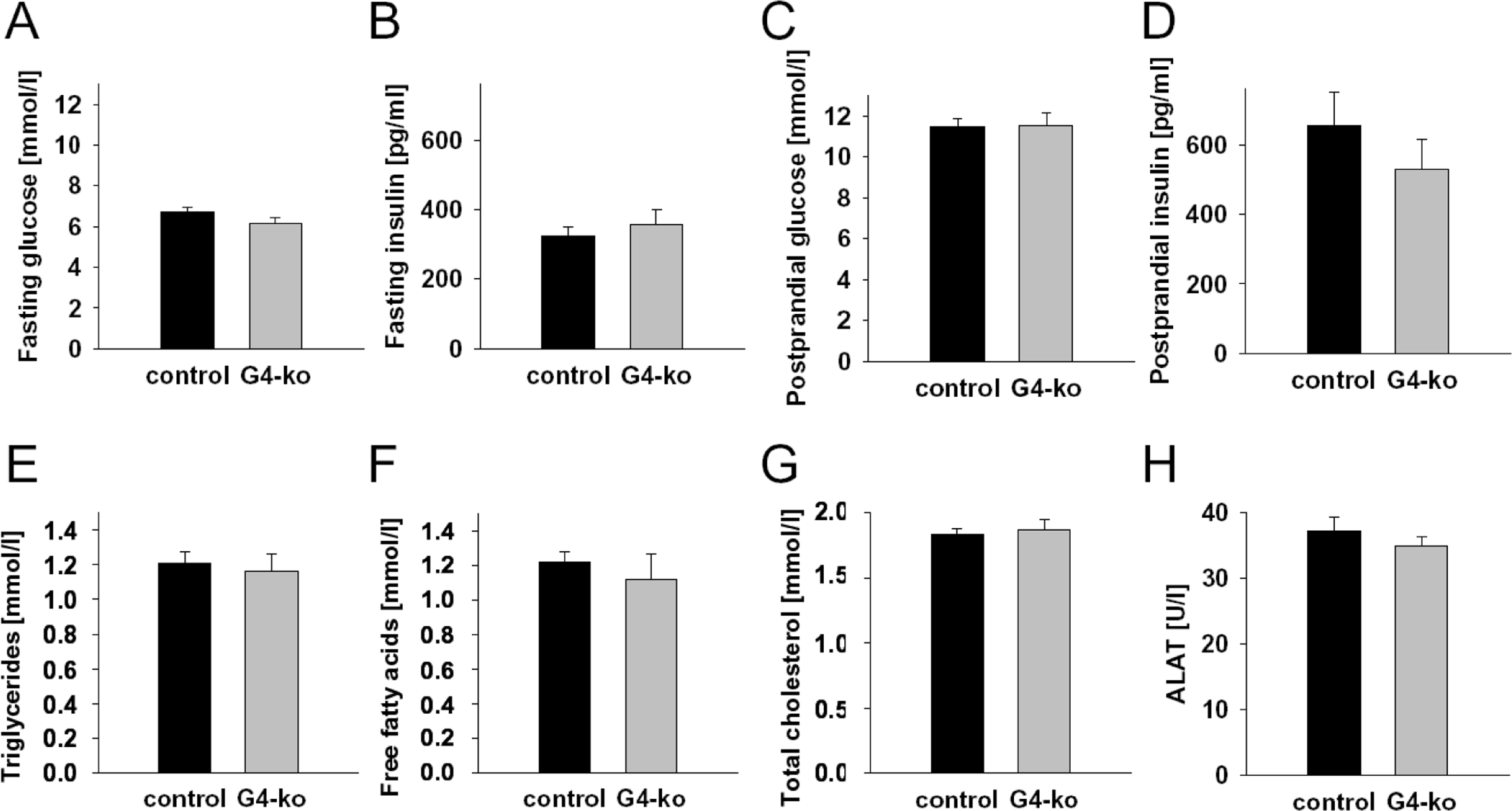

Supplement: Effects of impaired telomerase activity on metabolic serum markers. — (A, B) Fasting plasma glucose (A) and insulin (B) levels of mice that were food deprived for 16 hrs (C, D) Postprandial plasma glucose (C) and insulin (D) levels of mice that had ad libitum access to diet over night. (E to H) Fasting serum triglyceride (E), non-esterified fatty acid (F), total cholesterol (G) and alanine aminotransferase (ALAT) (H) levels. Black bars reflect control genotypes (n=42) and grey bars indicate Terc-/- G4 animals (n=19). [file aging-02-650-s001.tif]
